# Tum/RacGAP functions as a switch activating the Pav/kinesin-6 motor

**DOI:** 10.1038/ncomms11182

**Published:** 2016-04-19

**Authors:** Li Tao, Barbara Fasulo, Brandt Warecki, William Sullivan

**Affiliations:** 1 Department of Biology, University of Hawaii at Hilo, 200 West Kawili Street, Hilo, Hawaii 96720, USA; 2 Department of Molecular, Cellular and Developmental Biology, University of California, Santa Cruz, California 95064, USA

## Abstract

Centralspindlin is essential for central spindle and cleavage furrow formation. *Drosophila* centralspindlin consists of a kinesin-6 motor (Pav/kinesin-6) and a GTPase-activating protein (Tum/RacGAP). Centralspindlin localization to the central spindle is mediated by Pav/kinesin-6. While Tum/RacGAP has well-documented scaffolding functions, whether it influences Pav/kinesin-6 function is less well-explored. Here we demonstrate that both Pav/kinesin-6 and the centralspindlin complex (co-expressed Pav/Tum) have strong microtubule bundling activity. Centralspindlin also has robust plus-end-directed motility. In contrast, Pav/kinesin-6 alone cannot move microtubules. However, the addition of Tum/RacGAP or a 65 amino acid Tum/RacGAP fragment to Pav/kinesin-6 restores microtubule motility. Further, ATPase assays reveal that microtubule-stimulated ATPase activity of centralspindlin is seven times higher than that of Pav/kinesin-6. These findings are supported by *in vivo* studies demonstrating that in Tum/RacGAP-depleted S2 *Drosophila* cells, Pav/kinesin-6 exhibits severely reduced localization to the central spindle and an abnormal concentration at the centrosomes.

## Abstract (web summary)

Centralspindlin consists of dimeric kinesin-6 and dimeric RacGAP, and is involved in the organization of anaphase midzone microtubules. Here, the authors show that the RacGAP is needed for motor activity at the plus-end of microtubules, but not for the bundling activity associated with kinesin-6.

## Graphical abstract

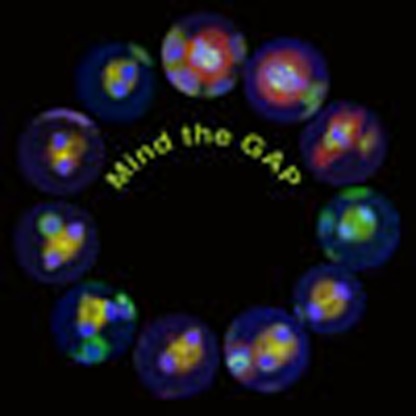

Centralspindlin is a heterotetrameric protein complex consisting of dimeric kinesin-6 (Pavarotti or Pav-KLP in *Drosophila melanogaster* (Pav/kinesin-6), ZEN-4 in *Caenorhabditis elegans* and CHO1/MKLP1 or MKLP2 in mammals) and the dimeric Rho-family GTPase-activating protein RacGAP (Tumbleweed or RacGAP50C in *D. melanogaster* (Tum/RacGAP), CYK-4 in *C. elegans* and MgcRacGAP in mammals)^1^,^2^,^3^,^4^. Centralspindlin was first identified for its role in organizing anaphase midzone MTs and cleavage furrow establishment^5^,^6^. Subsequent studies demonstrated that centralspindlin is also required for other MT-based processes including intracellular bridge formation, axon migration and establishment of embryonic anterior–posterior polarity^7^,^8^,^9^,^10^. In each of these instances, the MT-based motor activity is essential. Proper regulation of Pav/kinesin-6 motor activity is especially crucial for cytokinesis, because centralspindlin controls contractile ring timing and position.

RacGAP, with its well-characterized contractile ring-binding partners including RhoGEF, Aurora B kinase, kinesin-6 and Anillin, functions as a scaffold linking structural and regulatory furrow components to Pav/kinesin-6 for delivery to the site where the cleavage furrow forms^11^,^12^,^13^. While studies have provided evidence for MT plus-end-directed motor activity for centralspindlin^14^,^15^,^16^,^17^, it remains unclear whether RacGAP influences this activity. *In vivo* studies of *Drosophila* cytokinesis reveal that Pav/kinesin-6 fails to localize to anaphase midzone MT bundles in Tum/RacGAP mutants^11^. This suggests that Tum/RacGAP may promote Pav/kinesin-6 motor activity. However, recent studies suggest that RacGAP decreases motor activity, perhaps through RacGAP-induced conformational changes^16^,^17^.

The role of RacGAP in modulating kinesin-6 motor activity has remained unsolved in large measure due to difficulties in expressing and purifying soluble full-length kinesin-6 and RacGAP. Here by developing conditions to stably express full-length *Drosophila* Pav/kinesin-6 and Tum/RacGAP, we demonstrate that Tum/RacGAP is required for the plus-end-directed motor activity, but not the bundling activity of Pav/kinesin-6. In accord with this result, we find the ATPase activity of centralspindlin is many fold higher than that of Pav/kinesin-6 alone. Finally, our biochemical studies are supported by *in vivo* analysis. In Tum/RacGAP RNA interference (RNAi)-depleted S2 cells, Pav/kinesin-6 fails to concentrate at the midzone microtubules and instead maintains an unusually pronounced concentration at the centrosomes.

## Results

### Purification of centralspindlin components

**Figure 1:**
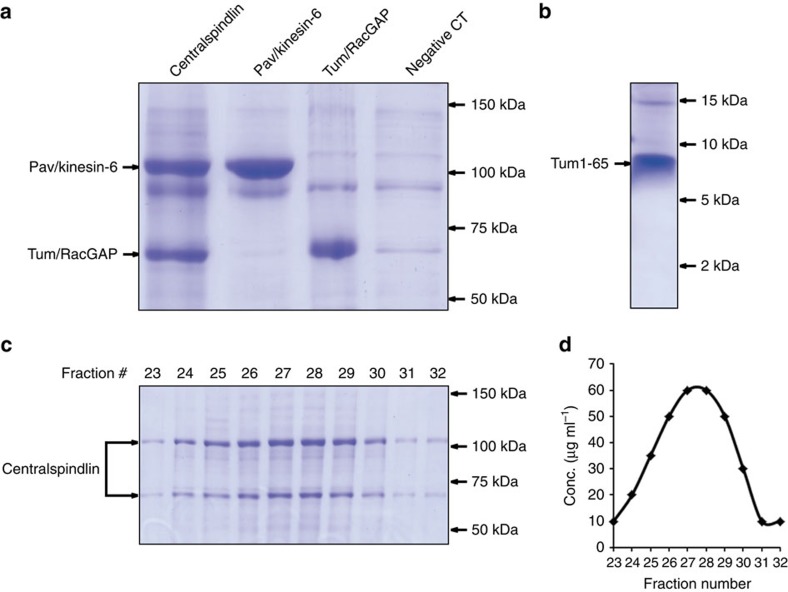
Purification of centralspindlin components from the baculovirus expression system. (**a**) Commassie blue-stained SDS–polyacrylamide gel (8%) shows eluates of full-length centralspindlin, Pav/kinesin-6, Tum/RacGAP and a negative control (protein purified from sf9 cells infected with no-gene-bearing baculoviruses) obtained from Ni-NTA affinity column. (**b**) Commassie blue-stained SDS–polyacrylamide gel (20%) shows fragment Tum1–65 eluate obtained from Ni-NTA affinity column. (**c**) Commassie blue-stained SDS–polyacrylamide gel shows the composition of protein fractions obtained during the purification of centralspindlin from the gel-filtration chromatography (Superose 6 FPLC, GE Pharmacia). (**d**) Corresponding plot of protein concentration versus fraction number demonstrates the centralspindlin complex elutes in a single peak.

The baculovirus-expressed *Drosophila* proteins Pav/kinesin-6 and Tum/RacGAP containing C-terminal 6 × His tags were purified separately from sf9 cell lysate with Ni-NTA affinity chromatography. For centralspindlin, viruses of C-terminal 6 × His-tagged Pav/kinesin-6 and viruses of non-tagged Tum/RacGAP were co-infected into sf9 cells. Ni-NTA affinity chromatography pulled down the centralspindlin complex (Fig. 1a), which suggests that Tum/RacGAP and Pav/kinesin-6 assemble into a centralspindlin complex inside sf9 cells. This interpretation is supported by gel-filtration chromatography studies (Superose 6, GE) in which Pav/kinesin-6 and Tum/RacGAP purified from co-infected cells simultaneously elute immediately after the void volume in a single peak (Fig. 1c,d). Hydrodynamic studies demonstrate that the recombinant centralspindlin has a heterotetrameric structure, while both Pav/kinesin-6 and Tum/RacGAP have homodimeric structures (Table 1). To exclude the possibility that non-specific contamination might contribute to activity in the following motor activity assays, proteins purified from sf9 cells that were infected with baculovirus bearing no gene (that is, virus generated from blue colonies) served as a negative control (Fig. 1a).

### Centralspindlin and Pav/kinesin-6 alone bundle MTs

**Figure 2:**
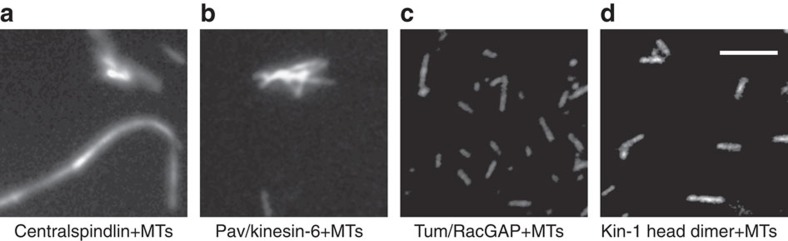
Purified centralspindlin and Pav/kinesin-6 both bundle MTs. Fluorescence microscopy demonstrates that purified centralspindlin (**a**) and Pav/kinesin-6 (**b**) have obvious bundling activity, while Tum/RacGAP (**c**) or kinesin-1 head dimer (**d**) cannot bundle MTs under the same conditions (1 mM ATP and 150 mM KCl). Scale bar, 10 μm.

A proposed function of centralspindlin is to organize the central spindle by concentrating at the spindle midzone and crosslinking anti-parallel MTs. To test this, full-length Pav/kinesin-6, Tum/RacGAP or centralspindlin were mixed with Rhodamine-labelled MTs under physiological ATP and salt conditions (1 mM ATP and 150 mM KCl). Fluorescent-based bundling assays revealed that as expected and previously reported^18^, the centralspindlin complex has strong MT-bundling activity (Fig. 2a). In three independent experiments, we observed bundling in all of the 36 slides examined. Surprisingly, we also found that Pav/kinesin-6 alone had robust bundling activity (Fig. 2b). In three independent experiments, we observed bundling in all of the 36 slides examined. In contrast, under the same conditions, we never observed MT bundling with Tum/RacGAP (Fig. 2c; 33 slides examined).

To determine if the intrinsic bundling ability of Pav/kinesin-6 might be a result of the kinesin head domains splaying apart and binding adjacent MTs, we assayed the MT-bundling activity of a control dimeric kinesin-1 neck-motor domain construct (Cytoskeleton Inc.). In three independent trials examining a total of 30 slides, we never observed bundled MTs (Fig. 2d). These data suggest, but do not prove, that Pav/kinesin-6 may have a domain, in addition to the head domain, that binds MTs.

The other possibility is that, just like muscle myosins which crosslink actin filaments and exert force by forming polymers^19^, Pav/kinesin-6 or centralspindlin form polymers on binding onto MTs. These results also indicate that Pav/kinesin-6 plays a key role in associating and assembling MTs to form the central spindle.

### Centralspindlin complex moves MTs in gliding assays

**Figure 3:**
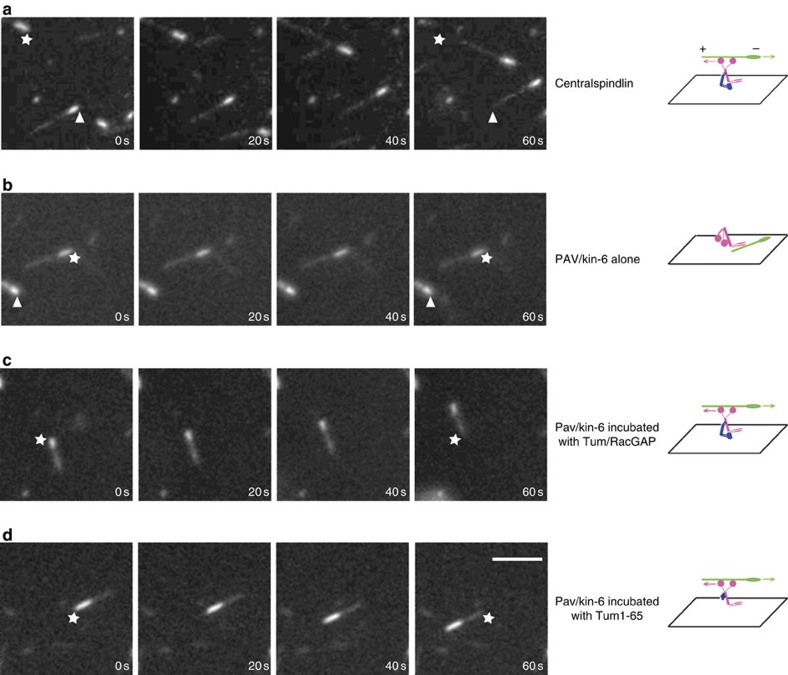
MT gliding assays reveal Tum/RacGAP is required for the motor activity of Pav/kinesin-6. Assays of MT gliding driven by (**a**) centralspindlin, (**b**) Pav/kinesin-6, (**c**) Pav/kinesin-6 incubated *in vitro* with Tum/RacGAP, (**d**) Pav/kinesin-6 incubated *in vitro* with Tum1–65 fragment. In each row of panels, stars and arrowheads are positioned in the same spot to judge movement. Polarity and direction of movement is indicated by a brighter minus-end. Scale bar, 5 μm.

**Figure 4:**
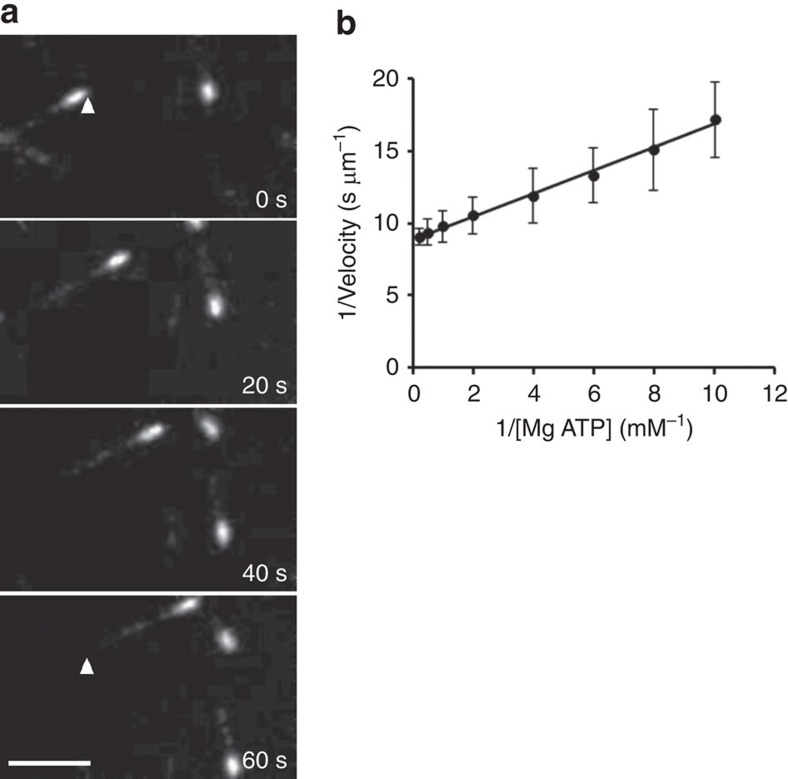
Characterization of recombinant centralspindlin. (**a**) MT gliding assay of purified centralspindlin. Fluorescence images of MTs moved by centralspindlin immobilized on a coverslip. Polarity-marked MTs (minus-end brighter) move in the gliding assays with velocities of 0.10 μm s^−1^. To judge movement, the arrowhead is positioned in the same spot in the first and last panels. MTs move with their minus-end leading, demonstrating that centralspindlin is a plus-end-directed motor. Scale bar, 5 μm. (**b**) Lineweaver–Burk plot displaying the effect of MgATP on MT motility. MT gliding velocities were measured in the presence of increasing MgATP concentrations and plotted as 1/velocity versus. 1/[MgATP]. This figure displays data from one experimental set, which had a measured apparent *K*_m_=0.089 mM for MgATP with a *V*_max_ of 0.11 μm s^−1^ (*r*^2^=0.99). Each point of 1/velocity was obtained from 15 independent MT moving events and given as average±s.d. in the plot.

Proposed activities for Pav/kinesin-6 include organization of the spindle by crosslinking and sliding anti-parallel MTs and regulation of cytokinesis by transport of regulatory components along MTs to the site of furrow formation^5^,^14^,^17^,^20^,^21^. However, direct analysis of Pav/kinesin-6 motor activity has not been tested with full-length purified proteins. Here we tested Pav/kinesin-6 motor function by using purified full-length proteins and *in vitro* MT gliding assays. Surprisingly, Pav/kinesin-6 does not move MTs under physiological ATP and salt conditions (Fig. 3b; See also Supplementary Movie 2), although it bound increasing numbers of MTs over time. Since Tum/RacGAP mutations disrupt Pav/kinesin-6 localization *in vivo*^7^,^18^,^22^, and this motor protein binds Tum/RacGAP to form centralspindlin, we tested if Tum/RacGAP binding is required for Pav/kinesin-6 motor activity. We repeated the MT gliding assays with Pav/kinesin-6 complexed with Tum/RacGAP (centralspindlin). Under identical ATP and salt conditions, we found that the centralspindlin complex has robust MT gliding motility (Fig. 3a and Fig. 4a; See also Supplementary Movie S1). Centralspindlin is plus-end directed as it moves MTs over the coverslips with the minus ends (brighter part) leading at 0.1 μm s^−1^ (Figs 3a and 4a and Table 2). To exclude the possibility that contaminants may be inducing the MT motility, a negative control assay was performed in parallel using a protein preparation from sf9 cells infected with no-gene-bearing baculoviruses (Fig. 1a).

To exclude the possibility that Pav/kinesin-6 loses its motility simply because the motor domain is stuck on the coverslip during the gliding assay, we performed an ‘antibody lifting assay', in which the coverslip is pre-treated with dimethyl-dichlorosilane (DDS) and the buffer is supplied with 0.2% Pluronic F127 (ref. 23). Under these conditions, centralspindlin cannot attach to the coverslip and glide MTs (Supplementary Movie 5). After anti-His antibodies were applied onto the coverslip to catch and lift 6His-tagged centralspindlin protein, centralspindlin is again capable of gliding MTs (Supplementary Movie 6). In contrast, antibody-lifted Pav/kinesin-6 alone still does not result in MT gliding (Supplementary Movie 7). Together, these results demonstrate that Tum/RacGAP is required for Pav/kinesin-6 motor activity.

To further characterize the centralspindlin-mediated MT motility, we measured MT gliding velocities as a function of MgATP concentration. The results show that when using ATP as substrate, centralspindlin-based MT motility obeys saturable Michaelis–Menten kinetics with an apparent *K*_m_ of 0.079 mM and *V*_max_ of 0.11 μm s^−1^ (Fig. 4b and Table 3). We also performed the same motility assay on purified Tum/RacGAP, and, as expected, no motility was observed.

### Addition of Tum/RacGAP promotes Pav/kinesin-6's activity

To further test the requirement of Tum/RacGAP for the Pav/kinesin-6 motor function, we performed an ‘add-back' assay. Gliding assays were performed on equal molar solutions of Pav/kinesin-6 and Tum/RacGAP proteins that were mixed and incubated *in vitro*. Hydrodynamic assays show that Pav/kinesin-6 and Tum/RacGAP are assembled into a heterotetramer, comparable to the baculovirus-expressed centralspindlin (Table 1). This *in vitro* ‘add-back' protein combination efficiently moves MTs in the gliding assay, similar to the centralspindlin holoenzyme purified from sf9 cells (Fig. 3c and Table 2; See also Supplementary Movie 3). In contrast, adding the negative control protein preparation (from sf9 cells infected with no-gene-bearing baculoviruses) into Pav/kinesin-6 does not restore MT motility. These results demonstrate that Tum/RacGAP and Pav/kinesin-6 can self-assemble into a functional centralspindlin complex without additional enzymes or chaperonins. They also demonstrate that Tum/RacGAP is required for Pav/kinesin-6 motor activity.

### Centralspindlin and Pav/kinesin-6 ATPase activity

Kinesin motors have intrinsic MT-stimulated ATPase activities. The force of kinesin to move MTs is generated from the hydrolysis of ATP. To test if Tum/RacGAP increases Pav/kinesin-6 motor activity through the increase of ATPase activity, we performed ATPase assays using the following combinations of centralspindlin components: centralspindlin complex, Pav+Tum1–65 fragment (rationale for this fragment is explained in next paragraph), Pav/kinesin-6, Tum/RacGAP and Tum1–65 fragment. The results show that the ATPase activity of centralspindlin or Pav+Tum1–65 fragment is seven times higher than Pav/kinesin-6 alone (Table 4). As expected, neither full-length Tum/RacGAP nor the Tum1–65 fragment exhibits ATPase activity (Table 4). This result supports our finding that Tum/RacGAP promotes Pav/kinesin-6's motor activity.

Previous studies demonstrated that some kinesins also use GTP for MT-based motility, although with a relatively lower affinity than ATP^24^,^25^. We were particularly interested in whether this was true for centralspindlin because Tum/RacGAP is associated with a number of binding partners, including Pebble/RhoGEF, the GTP exchange factor responsible for locally activating cortical RhoA^11^,^22^,^26^. Given Tum/RacGAP is the component of centralspindlin that binds and transports Pebble/RhoGEF^22^, it is possible that it may regulate Pav/kinesin-6 motor activity by recruiting and concentrating GTP and thus stimulating the motor's potential GTPase activity. However, we find that, unlike other kinesins^24^,^25^, centralspindlin cannot use GTP as a substrate to drive MT motility (Table 3). This result suggests that Pav/kinesin-6 has an unusually high specificity for ATP. Perhaps this is an adaptation preventing activation of the Pav/kinesin-6 motor in regions of highly localized concentrations of GTP.

### Tum1–65 fragment activates the motility of Pav/kinesin-6

We next determined which domain of Tum/RacGAP is primarily responsible for activating Pav/kinesin-6 motor activity. Previous work demonstrated that an N-terminal fragment of RacGAP binds the neck region and dramatically influences kinesin-6 conformation^11^,^17^,^27^. These findings made this fragment an especially promising candidate for modulating Pav/kinesin-6 motor activity. Consequently, we baculoviral-expressed and purified the corresponding 65 aa fragment (Fig. 1b) and performed the *in vitro* ‘add-back' assay by adding it to Pav/kinesin-6. This small peptide restores the Pav/kinesin-6 plus-end-directed MT motility (Fig. 3d, Table 2, see also Supplementary Movie 4). This result suggests that Tum/RacGAP activates the motor function of Pav/kinesin-6 by providing structural support and potentially changing the position or conformation of the motor head.

### Tum/RacGAP is required for Pav/kinesin-6 localization

**Figure 5:**
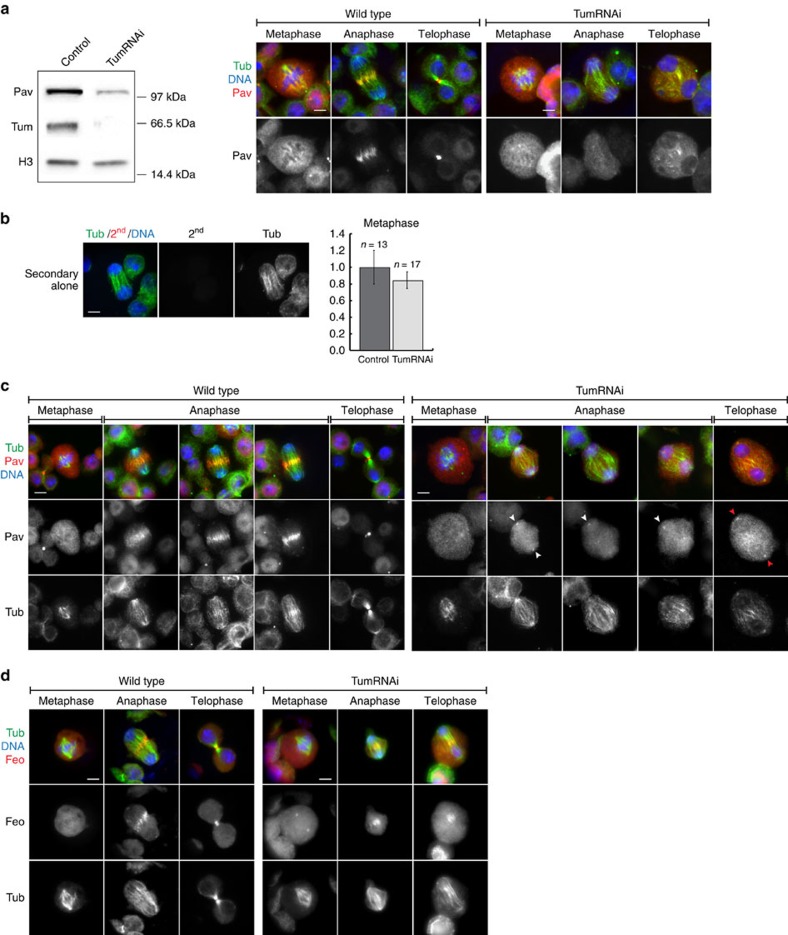
*In vivo* analysis reveals that Tum/RacGAP is required for Pav/kinesin-6 localization. (**a**) Left: western blot of Tum/RacGAP and Pav/kinesin-6 proteins in control and in Tum/RacGAP-depleted S2 cells. H3 protein used as a control. Right: Pav/kinesin-6 levels in Tum/RacGAP-depleted and control S2 cells. Images captured with the same exposure time. Scale bar, 5 μm (**b**) Left: auto-fluorescence test of secondary antibody used against Pav/kinesin-6. S2 cells stained with DAPI (blue), Tubulin (green) and secondary antibody (red) without anti-Pav/kinesin-6 primary antibody. Right: average fluorescence intensity of Pav/kinesin-6 normalized to the control in Tum/RacGAP-depleted cells during metaphase. (**c**) Metaphase, anaphase and telophase in control (left panel) and Tum/RacGAP-depleted (right panel) S2 cells. White arrowheads: localization of Pav/kinesin-6 at centrosomes of early-late anaphases. Red arrowheads: Pav/kinesin-6 localization on telophase centrosomes. Cells stained for tubulin (green), DNA (blue) and Pav/kinesin-6 (red). Scale bar, 5 μm. (**d**) Feo/PRC localization during metaphase, anaphase and telophase in control (left panel) and Tum/RacGAP-depleted (right panel) S2 cells. Cells stained for tubulin (green), DNA (blue) and Feo/PRC (red). Scale bar, 5 μm.

To investigate whether the above *in vitro* findings apply *in vivo*, we used RNA interference to strongly reduce Tum/RacGAP protein levels in *Drosophila* S2 tissue culture cells (Fig. 5). This cell line has been extensively used in functional studies of cytokinesis^28^,^29^,^30^. western blots demonstrate a dramatic decrease in Tum/RacGAP protein levels in Tum/RacGAP double-stranded RNA (dsRNA)-treated cells (Fig. 5a). As previously reported^31^, we find loss of Tum/RacGAP also results in a reduction in Pav/kinesin-6 levels. However, significant levels of Pav/kinesin-6 remained in the Tum/RacGAP-depleted cells (Fig. 5a). To determine that the western blot signal observed in Tum/RacGAP RNAi-treated cells was specific to the Pav/kinesin-6 primary antibody and not the result of non-specific binding or auto-fluorescence of the secondary antibody, we performed control experiments using the fluorescently-labelled secondary but not anti-Pav primary antibody (Fig. 5b). These studies clearly indicate that the Pav immunofluorescent signal observed in the Tum/RacGAP-depleted cells is neither the result of non-specific nor background fluorescence.

To determine if the reduced levels were still sufficient to evaluate Pav/kinesin-6 localization patterns in wild-type and Tum/RacGAP-depleted cells, we performed quantitative immunofluorescence. During metaphase in control cells, Pav/kinesin-6 is diffusely localized in the cytoplasm. Therefore, we focused our quantification on metaphase cells (Fig. 5b). As shown in Fig. 5a, cytoplasmic Pav/kinesin-6 is clearly visible in the Tum/RacGAP-depleted cells. In addition, we clearly detect ectopic centrosomal localization of Pav/kinesin-6 in the Tum/RacGAP-depleted cells (red-arrows Fig. 5c). Quantification of immunofluorescence in Tum/RacGAP-depleted cells also revealed a reduction in Pav/kinesin-6 levels although not as dramatic as observed by western analysis (Fig. 5b). One possibility for this difference is that the western quantification is based on cell numbers, and Tum/RacGAP-depleted cells have disrupted cytokinesis, resulting in multinucleate cells with altered cell volumes making a direct comparison to wild-type cells difficult.

Analysis of wild-type cells shows that during metaphase Pav/kinesin-6 is diffusely localized throughout the cytoplasm (*n*=13 (100%); Fig. 5c, left panel). In early and mid anaphase of wild-type cells, Pav/kinesin-6 rapidly accumulates on the overlap microtubules at the cell equator, forming a wide cortical band (*n*=16 (100%)). During late anaphase and telophase, the Pav/kinesin-6 band narrows concentrating at the plus-ends of central spindle overlapping microtubules (*n*=16 (100%)). These findings are consistent with previous descriptions of Pav/kinesin-6 localization throughout the cell cycle^5^,^29^,^32^.

We next examined Pav/kinesin-6 localization in cells treated for 72 h with Tum/RacGAP dsRNA to induce Tum/RacGAP decrease (Fig. 5c left panel). As observed for wild-type cells, Pav/kinesin-6 is diffusely localized throughout the cytoplasm during metaphase (*n*=17). In early and mid anaphase, spindle morphology and sister chromosome separation are normal in the Tum/RacGAP-depleted cells. However, the dramatic relocalization of cytoplasmic Pav/kinesin-6 to the midzone microtubules fails to occur in all cells examined (*n*=20). In addition, Pav/kinesin-6 often exhibits a distinct concentration at the centrosome in the Tum/RacGAP-depleted cells (15 out of 20 (75%) anaphases and 13 out of 18 (72%) telophases; Fig. 5c, white arrowheads).

Significantly, Pav/kinesin-6 localization to the early anaphase central overlap microtubules fails even though spindle morphology is normal (Fig. 5c, and Supplementary Fig. 1). As the Tum/RacGAP-depleted cells progress through anaphase and into telophase, while overlap midzone microtubules are present they become disorganized and a normal central spindle does not form (*n*=38 (100%); Fig. 5c).

To determine whether Tum/RacGAP depletion results in a general disruption of central spindle components and is therefore not specific to its effect on Pav/kinesin-6, we analysed the localization of Fascetto (Feo)/PRC^33^, a core central spindle component, during anaphase/telophase in wild-type and Tum/RacGAP-depleted cells. As shown in Fig. 5d, in wild-type cells Feo localizes in the cytoplasm during metaphase. While the cell progresses into anaphase, Feo localizes in a distinct band across the central spindle. During telophase, this band narrows as the central spindle contracts. During metaphase in Tum/RacGAP-depleted cells, Feo exhibits a distinct concentration at the centrosome, as well as the cytoplasmic localization observed in wild-type cells. During anaphase in Tum/RacGAP-depleted cells, Feo forms a distinct band at the central spindle although not as organized as the Feo band observed in wild-type cells. During telophase, there is a distinct narrow band of Feo, although much less compact and organized as seen in wild-type telophase cells. Thus, unlike Pav/kinesin-6, Feo is still able to localize to the central spindle in Tum/RacGAP-depleted cells. Together these results suggest that Tum/RacGAP depletion causes a specific disruption of Pav/kinesin-6 function.

## Discussion

Centralspindlin plays a key role in a number of events throughout cytokinesis. Early in the process, this complex is required for bundling MTs and central spindle formation. Later it provides a platform for the transport of key furrowing components such as RhoGEF, Anillin and other proteins to the site of cleavage furrow formation^11^,^12^,^13^,^34^. During the final stages of cytokinesis, centralspindlin is essential for mediating abscission^35^. While there is much evidence that Tum/RacGAP has a linker/scaffold role for delivery of contractile components to the site of furrow formation, other roles remain less clear and may vary with cell type^36^. Here we have used a combination of *in vitro* biochemical assays and cytological analysis to determine the individual and collaborative roles of Pav/kinesin-6 and Tum/RacGAP in MT bundling, central spindle formation and motor activity.

During the anaphase–telophase transition, centralspindlin is required to bundle anti-parallel MTs, an early step in central spindle formation. To determine the role of individual centralspindlin components on MT bundling, we performed *in vitro* bundling assays with purified Pav/Kinesin-6 and Tum/RacGAP. We found that Pav/Kinesin-6 alone and centralspindlin, but not Tum/RacGAP, efficiently captured and bundled MTs. Our dimeric kinesin-1 motor-neck domain control experiment demonstrated that the Pav/kinesin-6-mediated bundling is unlikely due to splayed motor domains capturing and binding neighbouring MTs. More likely, the bundling is occurring through MT-binding sites at the tail, as well as the head motor domain of Pav/kinesin-6. This is the mechanism by which other motor proteins such as kinesin-1, kinesin-5 and kinesin-14 bundle MTs^37^,^38^,^39^. Thus, Tum/RacGAP may play more of a regulatory role rather than providing a role in MT bundling by direct binding. The crosslinking of Pav/kinesin-6 between anti-parallel MTs could function as a brake to antagonize the MT–MT sliding of kinesin-5 during anaphase B or the MT–MT sliding of kinesin-1 during post-mitotic neurite formation^31^.

Previous *in vivo* studies and the results presented here examining the effects of the *Drosophila* RacGAP mutant (*tum*) on cell division revealed that although central spindle formation was abnormal, robust arrays of bundled anti-parallel MTs formed between anaphase/telophase sister nuclei^11^. We believe bundling ultimately fails in the later stages of anaphase because in the absence of Tum/RacGAP, Pav/kinesin-6 recruitment and concentration at the midzone region fails.

Our findings differ from previous *in vitro* studies which concluded that centralspindlin bundled MTs, but that kinesin-6 or RacGAP added individually failed to bundle MTs^18^. A possible explanation for the different results is that our bundling assays were performed under the physiological concentration of ATP (1 mM), while previous work used the non-hydrolysable ATP analogue AMPPNP (5 mM). The latter induces non-reversible MT binding^40^. Use of this analogue may alter kinesin-6 conformation and binding abilities. In addition, we used *Drosophila* rather than *C. elegans* centralspindlin proteins and the different results may be due to species-specific differences in intrinsic Pav/kinesin-6 MT-bundling activity. As described in more detail below, the different findings also may be due to our use of baculoviral-expressed full-length proteins rather than bacterial expressed protein fragments.

In contrast to our finding that Pav/Kinesin-6 has intrinsic bundling activity, we find Pav/kinesin-6 motor activity requires a physical association with Tum/RacGAP. This conclusion is based on our *in vitro* gliding assays: intact centralspindlin but not Pav/kinesin-6 alone is capable of plus-end-directed MT motor activity with a *V*_max_ of 0.11 μm s^−1^ and a *K*_m_ of 0.079 mM (Table 3). Like other kinesins^24^,^25^, MT-stimulated ATPase activity of centralspindlin follows Michaelis–Menten kinetics (Fig. 4b, Table 3). However, unlike kinesin-1 or kinesin-2, which can also hydrolyse GTP to move MTs (although less efficiently), centralspindlin cannot utilize MgGTP to generate force for MT movement (Table 3). Given that the RacGAP component of centralspindlin associates with small G proteins (for example, RhoGEF), which may recruit and concentrate GTPs, the high specificity for ATP may be necessary to prevent inappropriate regulation of centralspindlin.

To determine the *in vivo* relevance of these results, we examined Pav/kinesin-6 localization in Tum/RacGAP-depleted cells. Previous fixed analysis in *Drosophila* demonstrated that in the absence of Tum/RacGAP, Pav/kinesin-6 central spindle localization fails^11^. However because the central spindle was not properly formed in the Tum/RacGAP mutants, the absence of Pav/kinesin-6 localization could be an indirect effect. Consequently, we analysed Pav/kinesin-6 localization in Tum/RacGAP-depleted *Drosophila* S2 cells in which we focused on early, as well as the late stages in anaphase. Normally, Pav/kinesin-6 localizes to the equatorial overlapping MTs during early anaphase. In Tum/RacGAP-depleted cells, the organization of the spindle and the equatorial overlap of MTs are normal during early anaphase. However, Pav/kinesin-6 localization to the equator fails. We conclude the failed localization of Pav/kinesin-6 is not an indirect effect of disrupted anaphase spindle. Given our *in vitro* results, a more likely explanation is that Tum/RacGAP is directly required for Pav/kinesin-6 motor activity. Taken together, the *in vivo* and *in vitro* results indicate Tum/RacGAP is required to activate Pav/kinesin-6 motor activity, adding to its well-established scaffolding role delivering RhoGEF and other proteins to the spindle midzone. By integrating scaffolding sites and Pav/kinesin-6-activating sites, Tum/RacGAP ensures that only intact centralspindlin associated with essential structural and regulatory furrow components is recruited to the site of contractile ring assembly at the spindle midzone.

These studies are not in accord with previous work concluding that RacGAP is a negative regulator of kinesin-6 motor activity^16^,^17^. These differences may be accounted for by the fact that we are the first to use full-length proteins for these assays, while previous studies used a kinesin-6 fragment without a tail domain. The tail domain may have important regulatory function. For example, full-length kinesin-1 acts very differently from a kinesin-1 fragment without a tail. The kinesin-1 tail domain is an inhibitory regulator of the motor domain, since the tail physically contacts the head and inhibits its ATPase activity^41^,^42^,^43^,^44^. This inhibition can be removed if the tail of kinesin-1 binds to a cargo, or if the tail is cut off. The full-length kinesin-1 binds microtubules and moves about 10 times less frequently and exhibits discontinuous motion compared with a truncated kinesin lacking a tail^45^. In addition, the kinesin-6 fragment from the previous work was purified from a bacterial expression system, thus the protein could lack proper post-translational modifications (for example, phosphorylations on the motor domain), which are critical for its proper function^46^. Finally, as mentioned above, species-specific differences in the activity of these proteins may also account for the conflicting results.

In *C. elegans*, binding of the CYK-4/RacGAP subunit induces a large-scale conformational change in the ZEN-4/kinesin-6 subunit^17^. The fact that Tum/RacGAP directly binds and activates Pav/kinesin-6 motor activity raises the possibility that Tum/RacGAP-induced changes in Pav/kinesin-6 conformation promote motor activity. Previous studies identified an N-terminal 65 amino acid domain of Tum/RacGAP that directly binds Pav/kinesin-6 (ref. 11). Notably, it binds the neck/linker region of kinesin-6 (ref. 18), which is a key region regulating conformational changes for kinesin or myosin movement^47^,^48^. Based on this work, we tested and discovered that this N-terminal fragment, when added to purified Pav/kinesin-6, was capable of activating Pav/kinesin-6 in our gliding assays. We suspect that the activation of Pav/kinesin-6 motor activity may be due to conformation changes induced by the 65 aa fragment. Support for this interpretation comes from our finding that the *S* values of Pav/kinesin-6 alone and the Pav/Tum1–65 fragment complex were 5.7S and 7.9S, respectively (Supplementary Table 1). Thus, regulation of Pav/kinesin-6 motor activity may be similar to the regulation of myosin motor activity. Myosin light chains activate myosin motor activity by engaging the neck region of the myosin^49^. Perhaps like kinesin-1 (refs 41, 42, 43, 44), kinesin-6 alone may have a self-inhibitory conformation in which the tail shields the ATPase activity of the motor domain. Similar to the activating mechanism of kinesin-1 (refs 50, 51), binding of Tum/RacGAP may disrupt the shielding effect of the tail and activate kinesin-6's motor activity.

Tum/RacGAP depletion in S2 cells resulted in an abnormal centrosomal concentration of Pav/kinesin-6. It may be that this localization reveals a previously undetected intermediate step in centralspindlin localization to the central spindle: centralspindlin is first recruited to the centrosomes, possibly along MTs emanating from the MTOC. This implies an association with dynein. This may be analogous to the interaction between motors of opposite polarity in axonal or intraflagellar transport, where retrograde transport of kinesin is achieved via its association with dynein^52^.

We suspect the centrosomal localization of Pav/kinesin-6 had not been previously observed because it rapidly transits from the centrosome to the central spindle. In the absence of Tum/RacGAP, the Pav/kinesin-6 motor is inactive and thus accumulates at the centrosome, perhaps via Dynein-mediated minus-end transport as described above. That a step in centralspindlin localization involves the centrosome readily explains the unusual localization pattern of this complex observed in some cell types. For example, during the cortical syncytial divisions of the early *Drosophila* embryo, Rappaport-like cytokinesis furrows, known as metaphase furrows, form between astral microtubule arrays emanating from centrosomes of neighbouring nuclei^53^,^54^. Studies reveal that centralspindlin concentrates at regions of astral microtubule overlap and this localization is essential for metaphase furrow formation^32^,^55^. Centralspindlin also localizes to the central spindle but furrows do not form there. Previous studies demonstrated that furrows do not form at the central spindle because a maternal variant of RhoGEF is recruited to the astral MTs but not to the central spindle^55^. While the ectopic centralspindlin localization at astral overlap microtubules of the syncytial *Drosophila* embryo readily explains the formation of furrows at these regions of overlap, the mechanism by which centralspindlin localizes to both, the central spindle and astral microtubules, remained unclear. Our finding that the centralspindlin complex initially localizes to the centrosome provides a straightforward explanation because the plus-end-directed motor activity of centralspindlin would result in the observed localization to both the overlap microtubules of the central spindle and overlap astral microtubules. Significantly, centralspindlin localization to astral MT plus-ends has been observed in other cell types and in experimentally induced monopolar spindles^56^,^57^.

Given these examples, it is interesting that in most cells centralspindlin is observed only at the central spindle and not on the astral microtubules. One possibility is that under normal conditions, the tips of the astral microtubules do not overlap with opposing astral microtubule tips and the centralspindlin complex motors off the microtubules into the cytoplasm. In contrast, centralspindlin motoring towards the central spindle would encounter anti-parallel overlap microtubules and be stabilized. Alternatively, there may be as yet undescribed factors at the centrosome that preferentially direct the centralspindlin complex towards the central spindle.

## Methods

### Protein preparation

cDNA templates of Pav/kinesin-6 and Tum/RacGAP (Open Biosystems/Thermo Scientific) were checked by sequencing before use. Three base changes on Tum/RacGAP cDNA were identified (739 C mutated into A; 1334A missed; 1390 C mutated into A), which cause missense mutations, ORF changes and nonsense mutations. These mutations were corrected with ‘QuikChange Lightning Multi Site-Directed Mutagenesis Kit' (Agilent). Four different constructs corresponding to full-length Pav/kinesin-6 with 6 × His on C terminus, full-length Tum/RacGAP with 6 × His on C terminus, 1–65aa of Tum/RacGAP (Tum1–65) with 6 × His on C terminus and untagged full-length Tum/RacGAP were generated by PCR then cloned into pDonR221 vector (Invitrogen). The ‘Bac-to-bac' baculovirus expression system (Invitrogen) was used to generate recombinant baculoviruses. Amplified viruses were used to infect sf9 cells. The proteins were purified from cell lysates with a Ni-NTA affinity column (Qiagen) followed by superose 6 gel-filtration FPLC (GE Pharmacia).

### Hydrodynamic assays

Recombinant protein molecular weight was calculated based on experimentally determined sedimentation coefficient (*S* value) and Stokes radius (Rs) by use of the method of Siegel and Monty^58^. Gel-filtration FPLC (Superose 6) was used to determine Rs. Recombinant proteins and six protein standards (Thyroglobulin 8.58 nm, Apoferritin 6.06 nm, β-Amylase 5.4 nm, Alochol dehydrogenase 4.55 nm, bovine serum albumin (BSA) 3.61 nm and Carbonic Anhydrase 2.01 nm) were applied separately to the column. A linear plot of −1/2 logKav versus Rs was generated to calculate Rs values. In addition, a 5–20% sucrose gradient was used to determine the *S* values of centralspindlin, Pav/kinesin-6, Tum/RacGAP and Pav+Tum1–65. A 0–20% sucrose gradient was used to determine the *S* value of Tum1–65. Recombinant proteins with five protein standards (Ovalbumin 3.7S, BSA 4.4S, Aldolase 7.3S, Catalase 11.3S, Thyroglobulin 19.4S) were centrifuged on gradients for 9 h at 55,000 r.p.m. at 4 °C (Beckman SW55Ti rotor). Standard curves were created of *S* value versus peak fraction number. Subunit molecular weight was determined based on standard curves of Rm (mobility of protein/mobility of bromophenol blue). Recombinant protein peak fraction numbers were calculated by immunoblotting and Coomassie staining.

### MT-bundling assays

Fluorescent MTs were polymerized by incubating 25 mM tubulin (Cytoskeleton Inc.)+1 mM rhodamine tubulin (Cytoskeleton Inc.) with 1 mMGTP and 10 mM taxol in BRB80 (80 mM K_2_PIPES,1 mM EGTA, 1 mM MgCl2)+10% glycerol at 37 °C for 35 min. Bundling assays were performed in buffer T (20 mM Tris, pH 8.0, 150 mM KCl, 2 mM MgCl_2_, 1 mM DTT, protease inhibitors) with 2.5 μM MTs, 0.2 μM motor proteins, 10 μM taxol and 1 mM ATP (final concentrations). The mixture was rocked at 22 °C for 20 min and transferred into a flow chamber with a DEAE–Dextran-coated coverslip such that the unstuck proteins were washed out. Bundling of fluorescent MTs was observed with an inverted widefield fluorescent microscope. Each experiment was repeated 3 times, and each time ⩾10 slides were checked to ensure consistent results.

### MT gliding assays

Polarity-marked MT-gliding over a casein-treated glass coverslip coated with motor proteins was performed in buffer T (20 mM Tris, pH 8.0, 150 mM KCl, 2 mM MgCl_2_, 1 mM DTT, protease inhibitors). Motor proteins were adsorbed onto the coverslip, and then MTs plus ATP in buffer were introduced to the motors. MTs bound to the adsorbed motors and were transported by them along the coverslip surface. Polarity-marked MTs were visualized by fluorescence microscopy gliding over the coverslip surface in the presence of 1 mM ATP. Images were analysed with Image J. Only MTs performing smooth movements (no pauses or collisions with other MTs) for >1 min were used for velocity measurements. For the measurement of centralspindlin kinetics, these same gliding assays were performed under the conditions of increasing MgATP (or MgGTP ) concentrations and saturated MTs. *V*_max_ and apparent *K*_m_ were determined by plotting 1/velocity versus 1/nucleotide (Lineweaver–Burk plot).

### ‘Add-back' assays

Equal molar solutions of Pav/kinesin-6 and Tum/RacGAP (or Tum1–65 fragment) were mixed and incubated at 22 °C for 20 min. The mixture was used for the MT gliding assay as described above.

### Antibody lifting assay

The assays were performed with DDS (Sigma)-treated coverslips using buffer T supplied with 0.2% Pluronic F127 (Sigma). The chamber was incubated with anti-His antibody (Rockland, 1:25 dilution) for 5 min. After rinsing the chamber with buffer, motor proteins (or other centralspindlin components) were flushed into the chamber and caught by the antibodies. Unbound proteins were washed out with buffer. Finally, an aliquot of fluorescent MTs together with 1 mM ATP in buffer was introduced into the chamber.

### ATPase assay

Kinesin ATPase assay kit (BK053, Cytoskeleton Inc.) was used to measure the ATPase activities. A standard phosphate curve (OD_650_ versus [Pi]) was generated first. For the ATPase assay, one aliquot of the centralspindlin components was mixed with MTs in a reaction buffer (supplied by kit). ATP was added to each reaction (0.3 mM final) and incubated for exactly 5 min. The reactions were terminated by adding CytoPhos (supplied by kit). Immediately after a 10-min incubation, the absorbance at 650 nm was measured. The standard Pi curve was used to calculate the amount of Pi from each ATPase reaction.

### dsRNA synthesis

The RacGAP cDNA clone RE37229 was ordered from the *Drosophila* Genomics Resource Center (DGRC). The gene sequence was amplified by PCR with primers containing a 5′ T7 RNA polymerase-binding site (5′- TAATACGACTCACTATAGGGAGG -3′) flanked by a gene-specific sequence: the sense and antisense gene-specific sequences and their nucleotide positions are: sense 5′- AACCACACCTTC -3′ and antisense 5′- TGCATATAGCGA -3′ located at nucleotide 1,184 and 2,034 positions, respectively. The PCR products were purified with the QIAquick PCR Purification Kit and used as a template to produce dsRNA with the MEGAscript T7 transcription kit (Life technologies). The RNA product was treated with DNase I to digest template DNA, extracted with phenol/chloroform, ethanol precipitated and resuspended in PBS in DEPC-treated water. To ensure that most of the RNA product was in a ds form, the RNA solutions were heated at 65 °C for 30 min and then slowly cooled to room temperature.

### Cell cultures and RNAi treatments

S2 cells derived from a primary culture of 20–24-h-old *D. melanogaster* embryos (Thermo Fisher), were cultured at 25 °C in Shields and Sang M3 medium (Sigma) supplemented with 10% heat-inactivated foetal bovine serum (Gibco). RNAi treatment was carried out according to the study by Somma *et al*.^29^. Cells were suspended in serum-free Shields and Sang medium at a concentration of 1 × 10^6^ cells per ml, and plated, 0.6 ml per well, in a two-well slide culture chamber (Lab-Tek). To perform RNAi, each culture was inoculated with 5 μg ml^−1^ of dsRNA. After 1-h incubation at 25 °C, 0.6 ml of medium supplemented with 15% foetal bovine serum was added to each well. Control cultures were prepared in the same way but without addition of dsRNA. Both RNA-treated and control cells were grown for 72 h at 25 °C and then processed for cytological analysis.

### Cytological analysis

Cells adherent to Lab-Tek chamber slides (177,380) were rinsed in PBS. The cells were treated with BRB80 buffer for 5 min and then fixed for 10 min with 3.7% Formaldehyde in BRB80 buffer. After washing the cells in PBS for 5 min, they were treated with PBST (PBS+0.1% Triton X-100) for 15 min. Finally, the cells were blocked for 20 min in PBST containing 3% BSA. Both control and Tum/RacGAP dsRNA-treated cells were stained with anti-Tubulin diluted 1:200 (S9026 Sigma T clone DM1α), anti-Pav/kinesin-6 diluted 1:200 (kindly provided by D. Glover) or anti-Feo diluted 1:100 (Feo, kindly provided by M. Gatti) antibodies. Immunostained preparations were analysed with a Zeiss Axioskop 2 plus fluorescent microscope equipped with an AxioCam HRm CCD camera and images were acquired with a Plan-NEOFLUAR × 100 1.30 oil objective and Axiovision 4.6.3 software (Zeiss). Photoshop CC and Illustrator CC (Adobe) software were used to prepare the images. To confirm that the Pav/kinesin-6 signal observed in control and Tum/RacGAP RNAi-treated cells was specific to Pav/kinesin-6 primary antibody and not the result of non-specific binding or auto-fluorescence of the secondary antibody, we stained the cells with the Alexa Fluor 594 goat anti-rabbit secondary antibody alone.

### Quantitative immunofluorescence microscopy

To compare the levels of Pav/kinesin-6 in control and Tum/RacGAP dsRNA-treated S2 cells during metaphase, images were captured using the same exposure time and processed and analysed at the same time under identical conditions. To quantify relative fluorescence intensities, different representative images from control and Tum/RacGAP dsRNA-treated cells were chosen from a single experiment and were treated identically in parallel as follows. First, the average fluorescence intensity within the perimeter of each cell was measured using Volocity 5.4 software (Perkin Elmer). Second, the average level of background fluorescence surrounding the cell was measured and the black point of each image was adjusted. Finally, the average values were recorded in Excel, normalized to the control values and plotted in Excel using s.d. for error bars.

### Western blotting

For western blotting, proteins were extracted from 1 ml of control and Tum/RacGAP dsRNA-treated S2 cells. Cells were harvested by centrifugation and pellets were lysed in 60 μl of Laemmli. Lysate was N_2_ frozen, boiled 5 min and 15 μl of each sample were electrophoresed on a 4–15% SDS–polyacrylamide Mini-PROTEAN TGX Precast gel (Bio-Rad), transferred to nitrocellulose overnight at 4 °C, and incubated with primary and horseradish peroxidase-conjugated secondary antibodies. Secondary antibodies were detected using the SuperSignal West Pico Chemiluminescent Substrate (Thermo Scientific) and the ChemiDoc MP imaging System from Bio-Rad. Antibodies used for the blot include rabbit anti-Tum/RacGAP (diluted 1:2,500) generated against our baculovirus-expressed Tum/RacGAP protein (Cocalico Biological Inc), rabbit anti-histone H3 (diluted 1:8,250, Abcam, ab1791) and rabbit anti-Pav/kinesin-6 (diluted 1:1,250).

## Additional information

**How to cite this article:** Tao, L. *et al*. Tum/RacGAP functions as a switch activating the Pav/kinesin-6 motor. *Nat. Commun.* 7:11182 doi: 10.1038/ncomms11182 (2016).

## Supplementary Material

Supplementary Information Supplementary Figure 1 and Supplementary Table 1

Supplementary Movie 1 MT gliding driven by centralspindlin. This corresponds to Fig. 3A.

Supplementary Movie 2 MT gliding driven by Pav/kinesin-6. This corresponds to Fig. 3B.

Supplementary Movie 3 MT gliding driven by Pav/kinesin-6 incubated in vitro with Tum/RacGAP. This corresponds to Fig. 3C.

Supplementary Movie 4 MT gliding driven by Pav/kinesin-6 incubated in vitro with Tum1-65 fragment. This corresponds to Fig. 3D.

Supplementary Movie 5 MT gliding assay of centralspindlin without antibody lifting in DDS and Pluronic F127 treated chambers (Control).

Supplementary Movie 6 MT gliding assay of antibody-lifted centralspindlin in DDS and Pluronic F127 treated chambers.

Supplementary Movie 7 MT gliding assay of antibody-lifted Pav/kinesin-6 in DDS and Pluronic F127 treated chambers.

## Figures and Tables

**Table 1:** Hydrodynamic properties of various centralspindlin components.

	***S*** **value (1 × 10**^**−13**^ **s)**	**Rs (nm)**	**MW**_**holo**_ **(k_D_)**	**MW**_**sub**_ **(k_D_)**	**Subunit#**
Pav/kinesin-6 alone	5.72	8.95	214	107	2
Tum/RacGAP alone	4.44	6.22	116	65	2
Centralspindlin from coexpression	8.70	10.23	372	107 (Pav)65 (Tum)	4 (2 Pav+2 Tum)
Centralspindlin from *in vitro* add-back	8.65	9.74	352	107 (Pav)65 (Tum)	4 (2 Pav+2 Tum)

MW, molecular weight.

**Table 2:** Summary of gliding assays under different motor conditions.

	**Centralspindlin**	**Pav/Kinesin-6**	**Tum/RacGAP**	**Pav+Tum**	**Pav+Tum1–65**
Velocity (μm s^−1^)	0.103±0.012	0	0	0.062±0.019	0.075±0.013
Direction	+end directed	NA	NA	+end directed	+end directed

NA, not applicable.

**Table 3:** Comparison of the kinetic parameters between centralspindlin and other kinesins.

**Centralspindlin**	Kinesin-1*	**Kinesin-2**†
*MgATP*		
*V*_max_=0.11±0.01 μm s^−1^	*V*_max_=0.56±0.10 μm s^−1^	*V*_max_=0.26±0.01 μm s^−1^
*K*_m_=0.079±0.013 mM	*K*_m_=0.063±0.034 mM	*K*_m_=0.28±0.05 mM
		
*MgGTP*		
*V*_max_=0	*V*_max_=0.43±0.08 μm s^−1^	*V*_max_=0.058 μm s^−1^
*K*_m_=NA	*K*_m_=1.9±0.8 mM	*K*_m_=5.5 mM

^*^Cohn *et al*.^24^.

^†^Pan *et al*.^25^.

**Table 4:** Comparison of ATPase activities between centralspindlin's components.

	**Centralspindlin**	**Pav+Tum1–65**	**Pav/Kinesin-6**	**Tum/RacGAP**	**Tum1–65**
ATPase (nmol of Pi per min per nmol of substrate)	148.4±10.1	151.1±17.4	21.8±8.4	0	0
