# Supplementary material for: Tum/RacGAP functions as a switch activating the Pav/kinesin-6 motor
Source: Nat Commun. 2016 Apr 19;7:11182. doi: 10.1038/ncomms11182 (PMC4838857; doi:10.1038/ncomms11182)
Supplement: Supplementary Information — Supplementary Figure 1 and Supplementary Table 1 [file ncomms11182-s1.pdf]

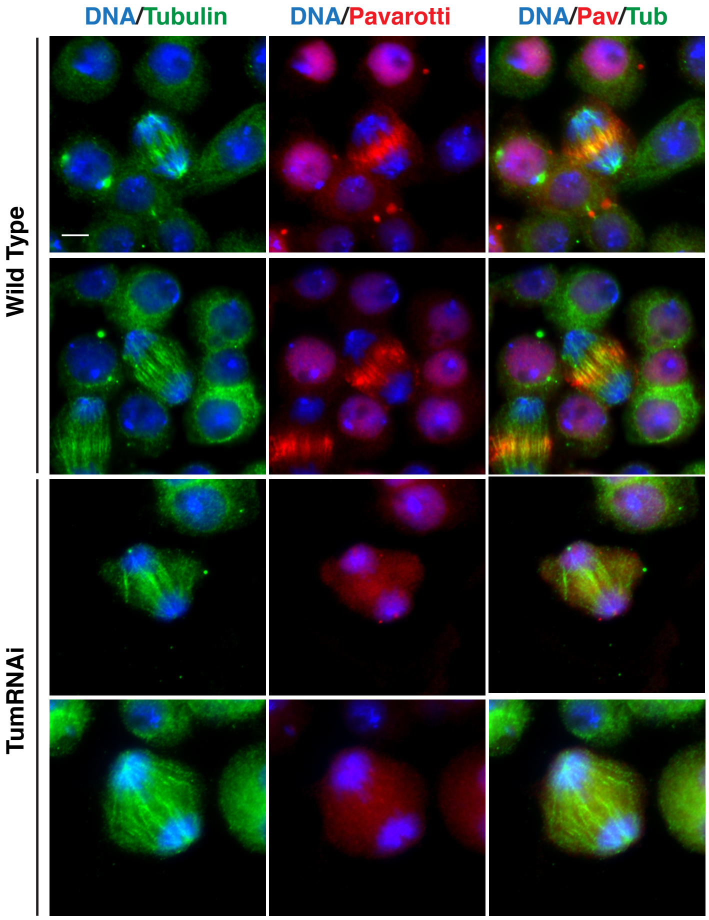

Supplementary Figure 1. Spindle organization during early anaphase in wild type and Tum/RacGAP dsRNA treated cells. The panel shows two wild type (rows 1 and 2) and two Tum/RacGAP dsRNA (rows 3 and 4) early anaphase figures. Pav/kinesin-6 antibody in red, tubulin antibody in green and DAPI in blue. Scale bar: 5 µm

Supplementary Table1. Comparison of sedimentation coefficients among centralspindlin's components.

	Centralspindlin	Pav/Kinesin-6	Tum1-65	Pav+Tum1-65
S value ($x10^{-13}$ s)	8.7	5.7	0.06	7.9
